# The Utility of the Koala Scat: A Scoping Review

**DOI:** 10.3390/biology13070523

**Published:** 2024-07-15

**Authors:** Stephen D. Johnston, Lyndal Hulse, Tamara Keeley, Albano Mucci, Jennifer Seddon, Sam Maynard

**Affiliations:** 1 School of Environment, The University of Queensland, Gatton 4343, Australia; l.hulse@uq.edu.au (L.H.); t.keeley@uq.edu.au (T.K.); a.mucci@uq.edu.au (A.M.); 2 School of Veterinary Science, The University of Queensland, Gatton 4343, Australia; jenny.seddon@jcu.edu.au; 3 Research Division, James Cook University, Townsville 4811, Australia; 4 Saunders Havill Group, Bowen Hills 4006, Australia; sammaynard@saundershavill.com

**Keywords:** koala, *Phascolarctos cinereus*, scat, non-invasive, methods, ecology, genetics, hormone metabolites

## Abstract

**Simple Summary:**

This review reports on the current and potential utility of the “koala scat” sample to provide a range of ecological and physiological assessments both at the population and individual animal level and do so in a non-invasive manner. DNA recovered from the scat sample provides useful information on koala distribution, diet, genetics and disease, whereas hormone metabolites can inform physiology. While there are still limitations with respect to the decay of quality DNA (host, microbiome, and pathogen) over time related to climate and sample handling, some of these issues can be overcome with timely sample collection. Other current limitations include an inability to detect and quantify particular hormone metabolites such as oestrogens and/or an appropriate biological interpretation of glucocorticoid metabolite secretion when measured in the faecal sample.

**Abstract:**

The use of samples or scats to provide important ecological, genetic, disease and physiology details on free-range populations is gaining popularity as an alternative non-invasive methodology. Koala populations in SE Queensland and NSW have recently been listed as endangered and continue to face anthropomorphic and stochastic environmental impacts that could potentially lead to their extinction. This scoping review examines the current and potential utility of the koala scat to contribute data relevant to the assessment of koala conservation status and decision making. Although we demonstrate that there is great potential for this methodology in providing details for both individual wild animal and population biology (distribution, abundance, sex ratio, immigration/emigration, genetic diversity, evolutionary significant unit, disease epidemiology, nutrition, reproductive status and stress physiology), the calibre of this information is likely to be a function of the quality of the scat that is sampled.

## 1. Introduction

Koala (*Phascolarctos cinereus*) populations are struggling to survive against the unyielding threats of habitat fragmentation, urban development, disease and stochastic catastrophic fires and drought associated with climate change. In 2022, the koala in Queensland, NSW and the ACT was listed as endangered under the Environment Protection and Biodiversity Conservation Act 1999 (EPBC Act) [1]. Despite conservation efforts over the last 20 years, koala numbers in SE Queensland and NSW continue to plummet, with the extinction of further local populations a real possibility. Compounding this bleak scenario, and particularly for the SE Queensland region, is the need to develop housing in order to accommodate an ever-increasing northward migrating human population. Queensland Government projections suggest a population density of 6 million people in SE Queensland by 2046, which represents an increase of nearly 160% on the current population numbers [2]; more people, means more housing supply, and even more pressure on threatened koala populations.

Clearly, regional planning will need to address, and if possible, attempt to mitigate this conflict. Koala management in South-east Queensland will not only require a thorough understanding of population abundance and distribution but also a much more comprehensive understanding of the current and predicted threats and stresses to these populations. Consequently, both natural and anthropomorphic threats on koala populations such as those imposed by climate change (bushfire and heat waves), disease (Chlamydiosis and Koala Retrovirus) and habitat fragmentation (housing, infrastructure, vehicle strike trauma, and dog attacks) will need to be carefully monitored and assessed for.

Methods for measuring koala population density and distribution have been evolving rapidly in the last decade such that there is now a wide range of protocols available for those conducting environmental assessments and impacts; these include direct koala spotting, the detection of koala scats (often assisted by tracker dogs), the location of animals in the canopy facilitated by infrared cameras mounted on drones, or a combination of all these approaches. Of these different methods, scat analysis offers a non-invasive and effective alternative to evaluating koala populations.

**Figure 1:**
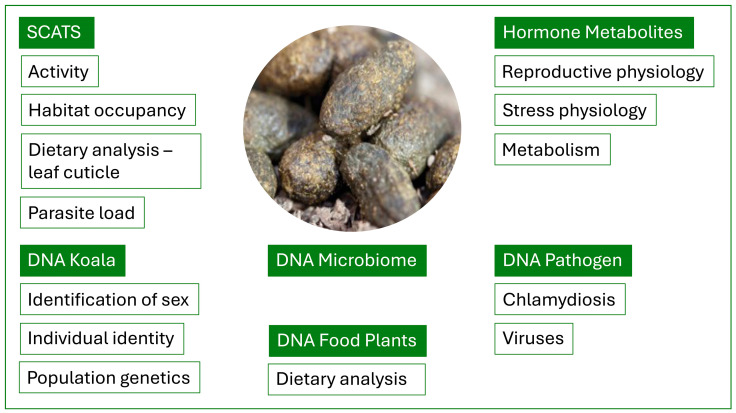
The “power” of koala poo—the information that is available from a koala scat.

An animal’s faecal sample or “scat” provides valuable non-invasive information on a range of biological processes and health status (Figure 1). Traditionally, this has included an analysis of diet and an indication of parasite load or disease, but with the further development of molecular and endocrine techniques, the scope of information can be dramatically expanded. When species defecate, they typically shed epithelial cells from the lower intestinal tract which deposit onto the surface of the scat as it is processed in the rectum. In herbivorous species that process their faeces into individual pellets, it is common to observe a mucous layer over the surface of the scat that contains epithelial cells, some of which possess intact nuclei that contain genetic information. Once these nucleated epithelial cells have been washed or scraped from the surface of the scat the DNA can be isolated. The DNA can then be used to confirm it has come from the host species and further analysed for genetic information via genotyping using markers such as microsatellites and SNPs. The resulting genetic data can provide a range of detail including the sex and genetic identity of the individual animal and how this genetic information might relate to other individuals within the population or to other surrounding populations.

The same molecular technology can be applied to the vegetal matter found in the pellet to further define what tree species the animal has been consuming and what gut microflora it might possess. The microbiome may also change between populations, time of the year, and perhaps even between leaves on the same tree. Molecular techniques can also determine whether the animal is shedding DNA from infectious pathogens (e.g., *Chlamydia* and retrovirus infection) and to quantify load. The use of faecal samples for determining endocrine detail has also become routine in the management and assessment of wildlife species health. The non-invasive nature of the sample collection and integrated secretion of the metabolites that are measured in the faeces overcome the confounding issue of the stress of taking a blood sample from a wild animal and the pulsatile secretion of hormones apparent in the systemic circulation [3]. Metabolites of hormones reflective of reproductive status, stress physiology and metabolism can all be measured in a faecal sample [4].

## 2. The Koala Scat

The koala scat has a characteristic morphology (Figure 1) and is therefore easily identified on the forest floor and persists in this form post-departure from the animal [5]. Koalas in captivity have been estimated to produce approximately 75–150 faecal pellets per day [6]. Locating koala faeces in the wild has also become increasingly more accurate and less labour intensive with the inclusion of detection dogs [7]. While the value of the faecal sample to koala monitoring has been postulated for decades, it has only been in the last 10 years that its utility for koala population assessment has become more fully realized [8]. The following review will examine the current use of faecal scat samples in koala conservation, highlighting the benefits and limitations of the information that can be obtained from its analysis. Unpublished data from our own ongoing studies will also be included for completeness.

## 3. The Non-Invasive Sample

The utility of sampling koala scats primarily stems from its collection as a non-invasively obtained biological sample. Particularly for the measurement of steroid hormones (e.g., stress and reproduction), obtaining a blood sample in most wildlife species is most likely to require the animal being captured and anesthetized or restrained, the act of which may potentially confound the very physiological parameter being assessed. The most obvious example of this would be the measurement of glucocorticoid hormones (cortisol and corticosterone) in a blood sample wherein the act of actively capturing and restraining a wild koala would most likely stimulate an acute stress response, negating its value as a reliable indicator of stress physiology both for the individual and the population [9,10]. Additionally, the measurement of hormone metabolites in the faeces typically represents an integrated sample of hormone secretion, thereby also overcoming any inherent episodic secretion of the hormone in the systemic circulation (e.g., testosterone) and negating the adverse effects on any altered hormone secretion associated with restraint or anaesthesia.

## 4. Habitat Occupancy and Activity

Currently, the most common application of scats in koala conservation are their use as an indirect measure for monitoring koala habitat occupancy. As a survey technique, the method is typically lower in cost to conduct and requires fewer resources than direct observation and is therefore popular for monitoring programs and environmental impact assessments [8]. Scat-based koala surveys have been used in Queensland since 1996 [11]. Standardized scat surveys include various versions of the Spot Assessment Technique [12], the Koala Rapid Assessment Method [5,7], and Balanced Koala Scat Survey [13].

Each method has its own inherent limitations in terms of accuracy and effort especially when used to extrapolate estimates of abundance, such that data collected from scat surveys should always be interpreted with caution [5]. False negative results can arise because of sampling technique but are also related to scat detectability, scat deposition, and decay rates that in turn can all vary between sites [8,14]. Cristescu et al. [7] have demonstrated that false negatives and survey time can be greatly reduced when detection dogs are used to locate scats; detection dogs are 19 times more efficient and 153% more accurate than humans at locating scats. It should be noted however that the use of detection dogs can be costly when compared to volunteers and requires the dog to be specifically trained. Nevertheless, scat surveys do have limitations; Ellis et al. [15] have indicated that scat searches are imprecise indicators of tree use. Scat surveys on their own also tell you nothing about the total number of animals, gender, health, diet, home range and movement patterns unless they can be supplemented with further laboratory analysis. Although scat surveys are likely to be suitable for determining koala activity levels or occupancy, alone they lack the robustness and accuracy as reliable estimates of density [8].

## 5. Dietary Analysis Using Cuticle Fragments

Koalas have been shown to exhibit degrees of preference for certain food species [16]. Food quality and availability are fundamental to quality koala habitat [17], so it is critical that we understand what koalas eat and why. The stomatal complex and arrangement of subsidiary and guard cells on the cuticle of *Eucalyptus* leaf fragments [18] that pass through into the koala scat have been used to determine dietary composition, and the technique found to be sufficient to separate out individual browse species [19,20]. The accuracy of this approach was examined by Ellis et al. [21] by feeding captive koalas with specified proportions of known browse species; this study revealed that the food species could be consistently detected in scat by microscopic analysis 34 h after the browse was first presented and that they remained in the scat for up to 154 h post-feeding. While the technique has subsequently been used by other researchers [22,23], the procedure is labour intensive, requires specific expertise and is unable to differentiate certain species of food trees [24].

## 6. Koala DNA Extraction from Faeces

The koala scat contains a range of host, dietary and pathogen DNA that can potentially be isolated via extraction and analysed in the laboratory to obtain a range of ecologically important information, particularly useful in assessing environmental impact [8]. The scat contains only very small amounts of koala host DNA recovered from epithelial cells exfoliated onto the surface of the faecal pellet, whereas the bulk of the DNA in the scat is derived from the various eucalypt species it consumes, the gut microbiota and/or pathogens. While scats processed for DNA dietary analysis typically utilize the whole sample, isolation of host DNA is best achieved by carefully lavaging off the outer mucous layer containing the host’s epithelial cells from the surface of the scat. The quantity and quality of the extracted DNA can then be assessed using spectrophotometry and confirmation of koala DNA achieved by reference to a house-keeping gene (e.g., Koala Beta actin).

Limitations with respect to this technique include ensuring that sufficient DNA is recovered for analysis [25], in addition to the presence of biological inhibitors, such as *Eucalyptus* tannins, which may interfere with downstream molecular analysis [26]. Wedrowicz et al. [25] have shown that the quality of the extracted DNA may also vary with commercial kits used; consequently, it is recommended that pilot studies first be conducted to assess and optimize the quantity and quality of the extracted DNA in advance of any genetic analysis. Schultz et al. [27] have indicated that genetic sampling is best undertaken with fresh faecal pellets (less than 2 days old) as the quality of extracted DNA from the scat declines over time; hence, the advantage of using scat detection dogs to ensure discovery of the freshest samples that might be overlooked beneath vegetation by humans. Wedrowicz et al. [28] have indicated that scats stored in paper bags or exposed to wet conditions (rain, condensation) may also result in lower DNA yields.

The downstream analysis of DNA isolated from scats, such as PCR and next-generation sequencing, can be challenging, as the extracted DNA is often fragmented and low in quality. Faecal DNA extracts contain a preponderance of DNA from exogenous (non-host) sources such as gut microbes, digesta, and environmental organisms. Gut bacteria pose a particular challenge as they account for the highest proportion of DNA in faeces. To address this issue, a method to enrich host DNA from non-invasive faecal samples may be employed. One such method utilizes natural differences in CpG-methylation density between vertebrate and bacterial genomes to preferentially bind and isolate host DNA from majority-bacterial samples, technically separating host DNA from exogenous DNA. Studies have demonstrated that host DNA enrichment from faecal scats is robust, efficient, and compatible with downstream molecular analysis [29].

## 7. Genetic Analysis

Population genetics is vital to understanding the long-term conservation management needs of any threatened wildlife species. At the population level, koala DNA isolated from scats has the potential to provide important detail, such as sex ratio and effective population size, genetic diversity, and migration (gene flow); it can also be used to better define what constitutes a metapopulation or evolutionary significant unit. At the individual animal level, genetic information obtained from scat DNA may be used to explore behavioural ecology (e.g., paternity, mating strategy, animal–animal relationships). Having the ability to genetically identify an individual koala (genetic profile) also allows this information to be used to determine habitat occupancy and/or activity and when used for mark—recapture studies, a potential estimate of abundance.

Recently, Hogg et al. [30] have systematically reviewed population genetic papers examining koala populations in eastern Australia from 1996 to 2020, noting that the majority of early studies utilized between 6 and 17 microsatellite genetic markers for their analyses. While microsatellite markers have had their place in koala genetic analysis, they are not representative of genome wide diversity and are currently being progressively replaced by the analysis of single nucleotide polymorphisms (SNPs). When compared to the koala reference genome [31], the discovery of SNPs obtained from next-generation sequencing can be used to determine genome wide diversity and/or explore the diversity in functional regions of the genome associated with different phenotypes.

As reviewed by Hogg et al. [30], there has been only a limited application of SNPs in studies of koala genetics to date [32,33,34]; while all these studies were conducted from DNA extracted from tissue, Schultz et al. [27] have also reported accurate SNP genotyping from koala DNA isolated from scats. The continuing decline in the cost of data acquisition of genomic studies will undoubtedly lead to the more widespread application of SNPs and/or whole animal genomic analysis, especially as the open database of the Koala Genome Survey continues to grow [30]. To date, 430 koala genomes have been released on Amazon Web Services Open Data program (https://awgg-lab.github.io/australasiangenomes/species/Phascolarctos_cinereus.html (accessed on 21 June 2024)). If scat DNA is to make the best use of this database, then the quality of faecal DNA extraction procedures will need to keep up. In addition, if we are to better understand the genetic connectivity of koala populations, we need to implement a coordinated approach to population genetic analysis via the availability of a standardized suite of publicly accessible genetic markers to all koala researchers and managers; this will facilitate an ability to directly compare the genetic health of regional koala populations.

## 8. Dietary Analysis Based on DNA

Molecular biology not only has an important role in koala genetics, but it can also be used to gain deeper insights into what koalas eat. Next-generation sequencing of *Eucalyptus* SNPs extracted from koala scat can be used as potential biomarkers of koala diet using a method known as DArTseq^TM^. Schultz et al. [27] demonstrated the potential of SNPs to be used in this way. More recently, Blyton et al. [24] have further refined this approach to focus on dietary species-specific SNPs using the plant DNA in the koala scat, resulting in a tool that allows a semi-quantitative analysis of what koalas eat. Blyton et al. [24] not only found general agreement with respect to what tree species koalas were already known to consume but also identified new species that could be contributing to their diet. This molecular approach to dietary analysis will facilitate the comparison of koala food species and preference between locations, season and perhaps, even of browse types consumed within the same tree.

## 9. Microbiome

The microbiome is the collection of all microbes that naturally live on or in the body and represents the primary interface between the organism and its environment. In addition to what the koala eats, molecular biology conducted on DNA isolated from the koala scat is being used to identify the microbial diversity and abundance of the koala gut microbiome, how it differs in various geographical locations and season, and how it changes over life history. Dietary focused information based on microbiome analysis is likely to have an important role in helping to make population management decisions such as recommendations on koala translocation. In a similar manner, analysis of changes in the microbiome is likely to have a therapeutic application following antibiotic treatment in order to prevent or manage dysbiosis.

Early studies used metagenomic analysis of 16S ribosomal RNA to profile the koala gut microbiome. Barker et al. [35] examined two koalas at different sites along the digestive tract (caecum, colon, and scat), reporting a highly complex and diverse ecosystem with considerable intra-individual variation. Later, Alfano et al. [36] also compared the microbiomes of two koalas from different regions of the body (eye, rectum, and scats), noting that the scat microbiome represented only a subset of that found in the rectum. Metagenomics was used by Shiffman et al. [37] to compare the respective microbiomes of the koala and wombat in order to contrast differences associated with dietary specialization; this analysis revealed that koala scat samples were dominant in the micro-organisms necessary for secondary metabolism, perhaps thereby indicating their role as an important player in the koala’s ability to detoxify its diet.

The faecal microbiome was further characterized in wild koalas by Brice et al. [38]; targeting the bacterial 16S ribosomal RNA (rRNA) gene, these researchers found a strong association between the microbial community and host diet and concluded that even amongst individuals that a change in consumption of congeneric tree species to another, can significantly alter the gut microbiome. This finding was further supported by that of Blyton et al. [39] who showed that koalas feeding on different tree species (i.e., Messmate and Manna Gum) had different microbiomes based on 16S rRNA profiles, despite also showing some overlap. Blyton et al. [39] also demonstrated that the microbiome of the koala could be altered by means of faecal inoculation. Translational studies of the koala scat microbiome are likely to follow in the future, including the use of faecal inoculation capsules to overcome antibiotic-induced gastrointestinal dysbiosis and their use in preparing koalas for translocation to assist individual koala gut microbiomes to adapt to shifts in diet [39]. Blyton et al. [40,41] have also examined changes in the scat microbiome to explore maturational changes between juvenile and adult assemblages of microorganisms associated with pap feeding in both captive and wild populations.

Also relevant to understanding the koala microbiome but not within the scope of this review is its potential role in facilitating chemical signals that might be used for reproduction, behaviour and communication.

## 10. Chlamydiosis

Apart from the threat of natural disasters (drought, heatwaves, and bushfires) and anthropomorphic disturbance (habitat loss, road, and dog trauma), koalas are also susceptible to debilitating diseases. *Chlamydia* spp. is an intracellular bacterium that causes significant inflammation of the conjunctiva and urogenital systems of both female and male koalas resulting in clinical blindness, cystitis, and infertility [42,43]. Although commonly associated with the urogenital tract and detection best sampled from swabs of this region, it is also possible to detect chlamydial DNA from faecal scat samples of infected individuals [44,45]. Using PCR, Wedrowicz et al. [44] reported a high concordance between *Chlamydia pecorum* in the DNA isolated from scats and urogenital swabs. Cristescu et al. [45] also compared the clinical efficiency (sensitivity and specificity) of a multiplex quantitative PCR (qPCR), next-generation sequencing (DArTseq^TM^) and a detection dog to correctly identify scats excreted by koalas that tested positive to *C. pecorum* from urogenital swabs and which showed observable clinical signs of the disease; all three methods showed 100% specificity but there was variable sensitivity (qPCR—78%; DArTseq^TM^—50%; detection dog—100%). The authors suggested that the lower sensitivity of the qPCR and DArTseq^TM^ compared to the detection was down to the fact that the molecular methods relied on the acquisition and extraction of quality DNA, whereas the dog was likely to be detecting volatile organic compounds released from the scat. Recent developments in the use of loop mediated isothermal amplification (LAMP) assays that can be readily conducted in the veterinary clinic and/or field [46,47] are providing a rapid diagnostic tool for detection of *Chlamydia* DNA from swabs but have yet to be developed for scat samples. It also needs to be remembered that current molecular methods for both swabs and scats are only detecting chlamydial DNA and are unable to distinguish live or infectious elementary bodies.

## 11. Viruses

Koala retrovirus is a gammaretrovirus that has been linked to koala neoplasia [48,49,50], with additional associations to immunosuppression [51,52,53]. To date, these links appear to be primarily correlative rather than causative [43]. Of all the KoRV subtypes, KoRV B is most commonly associated with lymphomas, leukaemias, and neoplasia [53,54]. The ability to detect KoRV A subtype and determine copy number in the koala scat was first reported by Wedrowicz et al. [44] using real time PCR. Quigley et al. [43] have used DNA extracted from scats to conduct a validation between sample type (blood, swab, and scat) in preparation for a phylogenetic and geographical analysis of KoRV subtypes, concluding a general agreement between combined diversity profiles for all tissue types (blood—100%, swab—98%, scat—90%). More recently, Blyton et al. [55] successfully validated and applied PCR and deep sequencing to DNA extracted from the koala scat to characterize KoRV A and a range of other exogenous subtypes (B-M) in a geographical study; this work confirmed that subtype A appears to have endogenized in northern koalas and is progressively becoming endogenized into the southern koala genome. The other exogenous subtypes of KoRV (B-M) detected by Blyton et al. [55] only appear to be found in northern koalas and were geographically restricted, suggestive of sporadic evolution and local transmission.

Recently, two gammaherpesviruses, phascolarctid gammaherpesvirus 1 (PhaHV-1) and phascolarctid gammaherpesvirus 2 (PhqHV-2), have been described in the koala, and while the pathogenicity of these viruses are still not understood, point of care assays have been successfully developed based on viral DNA extracted from koala scat samples [56].

## 12. Koala Reproductive Hormones

The measurement of hormone metabolites in faecal samples to assess reproductive status in wildlife has become increasingly common practice in eutherian [57,58,59] and to a lesser extent marsupial species [60]. Hormone metabolites are typically extracted from faeces using alcohol solvents and the reconstituted hormones are measured following biological validation via enzyme immunoassays using antisera with appropriate cross-reactivity. Reproductive steroid hormones such as progesterone, [61] and testosterone, [62] and their metabolites have been successfully analysed in koala faeces with the detection of the luteal phase and pregnancy in females and age and season changes in males. In fact, unpublished data from our group have shown how the measurement of progesterone metabolites recovered from the koala scat can be used to precisely map the luteal phase or pregnancy with the same degree of resolution as that measured in plasma samples [63]. We also have unpublished data on androgen secretion in the male koala, which has a direct application for the assessment of seasonality and sexual maturity.

However, despite numerous attempts using a range of different specific antibodies (e.g., oestradiol, oestrone-3-glucuronide) to analyse faecal extracts of koalas of known reproductive status (e.g., oestrus), and even when using multiple high pressure liquid chromatography (HPLC) and mass-spectrophotometry (MS) analyses, all attempts to detect biologically relevant oestrogen or oestrogen metabolite levels in koala scats have failed thus far. Hence, the measurement of oestrogen in faeces remains a significant challenge in the koala. Schwarzenberger et al. [57] have indicated oestrogen measurement in the faeces of herbivores can be problematic not only because of the low levels in the plasma (picograms rather than nanograms), but also because the main route of excretion is often via urine. An inability therefore to measure oestrogen readily in koala faecal samples means that we are currently limited to only identifying or monitoring animals that ovulate (luteal phase) or that are pregnant. Although reproductive steroids can be measured in faeces, it is not possible to measure protein-based gonadotrophins in the scat such as luteinizing hormone (LH) or follicle-stimulating hormone (FSH) as these molecules are excreted in the urine or denatured and degraded by the digestive system by the time they are excreted in the faeces [3].

## 13. Koala Glucocorticoid Hormones

Fanson et al. [64] have examined the utility of commonly used faecal hormone assays in marsupials and noted that the best performing assays varied with species, emphasizing the need to have thorough biological validation for each species and sample type. The measurement of glucocorticoid hormones (e.g., cortisol) from the koala scat has attracted significant attention in the literature (see Table 1 [64,65,66,67,68,69,70,71,72,73,74,75,76,77,78,79]). Numerous studies have attempted to validate the measurement of glucocorticoids (GC) using ACTH, exogenous cortisol or following a biological (physiological or behavioural) event in both plasma and faeces and to apply the assessment of faecal GC levels as an index of stress. One study incorporated liquid chromatography combined with mass spectrophotometry (LCMS) to specifically identify which glucocorticoid metabolites are present in the koala scat after the administration of exogenous cortisol [73]. GC metabolites in koala scats have been used to try to assess stress in both captive and wild populations including the effects of habitat fragmentation, translocation, disease, trauma, hospitalization and the handling of captive koala during for photography (Table 1). Parker-Fisher and Romero [80] highlighted the importance of using multiple measures of stress physiology (such as behaviour, immune function, health, weight changes), rather than restricting studies to single measures like GC, as this is likely to provide a better assessment of whether a species or individual is experiencing stress.

The interpretation of faecal GC measurements is challenging and open to debate given the significant variation that may occur in GC hormone secretion between individual animals, the role of GCs as mediators of the physiological stress response, as well as the maintenance of homeostasis and energy regulation, among others [81,82]. Acute stress responses are normal and typically do not have a long term or significant impact on the individual as GC levels and should quickly recover once they have served their role. Chronic stress responses may initially follow the same pattern as an acute response before GC dysregulation ensues (e.g., loss of normal feedback regulation); therefore, ensuring sample collection frequency and extent is adequate to distinguish between the two is essential. Equally challenging is the fact that there is no “typical” chronic GC production pattern; in response to a chronic stressor, GC production can increase, decrease or be unresponsive [81]. This emphasizes the need to integrate other measures of stress to properly evaluate the koala’s physiological state and response to the stressor of interest.

## 14. Koala Metabolic Hormones

Thyroid hormones are important for the regulation of metabolic activity in mammals and crosstalk with other hormone systems allows these hormones to coordinate metabolic changes and/or to modify the growth and maintenance of an organism with respect to environmental conditions [83]. Thyroid hormones, therefore, have the potential to serve as biomarkers for ecological studies, energy allocation and growth, and for the monitoring of physiological changes associated with food deprivation, food quality or reproduction. While no published studies currently exist for the measurement of nutritional faecal hormone metabolites in the koala, metabolites of thyroid (T3/T4) have been attempted in a range of eutherian wildlife species [83,84]. Our group is currently attempting to test and validate faecal T3 and T4 analysis techniques for the koala, and if successful, this could provide valuable physiological data for in situ research.

## 15. Implications: The Power of Koala Poo

This review has revealed the current and potential utility of a “koala scat” sample to provide a range of ecological and physiological assessments both at the population and individual animal level. While there are still limitations with respect to the decay of DNA (host, microbiome, and pathogen) and hormone metabolites over time, related to climate (e.g., rain, humidity, temperature) and sample handling, these issues may be overcome with the use of detection dogs in order to find freshly deposited scats.

The ability to identify individual koalas from scat DNA has a range of applications for researchers, site managers, and environmental assessment teams. It provides a powerful tool to obtain accurate estimates of population dynamics (size, sex ratio, birth, and death rate, immigration, and emigration rates), genetic diversity and habitat utilization and occupancy in a relatively short period of time. These data could be used to inform planning and environmental impact assessment processes providing a detailed and accurate picture of koala population dynamics and usage in areas identified for future housing or infrastructure development. With further improved methods of DNA isolation and the application of next-generation sequencing technology (SNPs and whole genome sequencing), it is not difficult to envision how scat DNA will also provide detailed information on behavioural ecology and disease susceptibility (e.g., MHC diversity). At the population level, scat DNA could be used to define evolutionarily significant units and as a tool to improve genetic management (e.g., translocation and genetic health). These techniques could be utilized in existing reserves and environmental offset sites to achieve improved outcomes for existing and new koala populations.

This review has also shown that disease (*Chlamydia* and KoRV subtype) in the population or individual koala can be assessed using scat DNA. The detection of *Chlamydia* DNA is already being utilized but is most likely to be that only associated with urogenital infection (*C. pecorum*). While quantitative PCR has the capacity to quantify *Chlamydia* load in order to make comparisons between populations, it is currently not possible to differentiate infectious elementary bodies from that of degraded *Chlamydia* DNA, so it is not possible to identify those koalas with an active infection from those who are shedding chlamydial elementary bodies. Nevertheless, scat DNA may still be particularly useful for scanning populations prior to translocation or determining the efficacy of *Chlamydia* vaccination programs. Studies using scat DNA to assess the population for the KoRV subtype has already provided valuable data on the epidemiology of the virus; an ability to identify KoRV in populations and individuals will also be important for disease management.

Studies of reproductive hormone metabolites from koala scats are highly instructive as markers for progesterone (ovulation and gestation) and testosterone secretion but further research is required to identify suitable analysis techniques that allow the successful monitoring of faecal oestrogens and the koala oestrous cycle. There has been a range of different studies validating suitable faecal glucocorticoid enzyme immunoassays to attempt to evaluate the impact of stressors in captive and wild koala populations. However, the interpretation of these hormone profiles requires further research, both in terms of the normative and abnormal responses to acute and chronic stress, individual animal variation and a better understanding of koala adrenal physiology. It will also be important to not rely on single measures of stress physiology (e.g., GC secretion) but rather combine these with other assessments (e.g., behaviour, health biomarkers). An ability to monitor koala metabolism through the measurement of faecal thyroid metabolites may allow managers to better assess the impact of reduced nutritional quality of eucalypt fodder associated with climate change (e.g., heat stress, increased sclerophylly and drought).

## Figures and Tables

**Table 1:** A Summary of GC studies using koala scats.

Metabolite	Type of Study	Assay	Reference
Cortisol	V—ACTH, Captive	EIA	[65]
Cortisol	A—Habitat (Aridity) (Semi-arid Zone), Wild	EIA	[66]
Cortisol	V—ACTH, A—Captive V Wild, Handling	EIA	[67]
Cortisol, CS	V—ACTH	EIA	[68]
Cortisol, CS, 72a, 37e	V—ACTH, Captive	EIA	[64]
Cortisol	A—Zoo visitor, Captive	EIA	[69]
Cortisol	A—Habitat clearing, Wild	EIA	[70]
Cortisol	A—Disease, Trauma, Hospital, Bushfire, Wild	EIA	[71]
Cortisol	A—Disease, Trauma, Hospital, Bushfire, Wild	EIA	[72]
Cortisol, 37e, 50c	V—Hydrocortisone, Captive	LCMS, EIA	[73]
Cortisol, 37e, 50c	V—Time decay, Water loss, Captive	EIA	[74]
Cortisol, 37e, 50c	A—Seasonality, Captive	EIA	[75]
Cortisol	A—Hospitalised, Rehabilitation	EIA	[76]
Cortisol	A—Translocation, Wild	EIA	[77]
Cortisol	A—Wild, Influence of cultural burns	EIA	[78]
Cortisol, 50c	A—Hospitalised	EIA	[79]

Key: A—Application, CS—corticosterone, EIA—Enzyme Immunoassay, LCMS—liquid chromatography mass spectrophotometry, ACTH—Adrenocorticotropic hormone, V—validation, 37e—5a-pregnane-3B,11B,21-triol-20-one, 50c—Tetrahydrocorticosterone, 72a—11-Oxoaetiocholanolone-3-HS.
